# Therapeutic Options for Patients who are not Eligible for Intensive Chemotherapy

**DOI:** 10.4084/MJHID.2013.050

**Published:** 2013-07-12

**Authors:** Elihu Estey

**Affiliations:** University of Washington and Fred Hutchinson Cancer Research Center, Seattle WA, USA

## Abstract

Although “less intense” therapies are finding more use in AML, the principal problem in AML remains lack of efficacy rather than toxicity. Hence less intense therapies are of little use if they are not more effective as well as less toxic than standard therapies. Assignment of patients to less intense therapies should be based on other factors in addition to age. Azacitidine and decitabine, the most commonly used less intense therapies in AML very probably produce better OS than best “supportive care” or “low-dose” ara-C. However improvement is relatively small when compared to expected life expectancy in the absence of disease. Accordingly, while azacitidine or decitabine should be considered the standards against which newer therapies are compared, continued investigation of potentially more effective therapies needs to continue. Better means for evaluating the large number of these therapies (and their combinations) are also needed.

## General Considerations

There has been recent emphasis on “less intense” therapy for AML. This emphasis might lead some to suspect that the principal cause of failure of AML therapy is treatment related mortality (TRM) due to use of intensive therapy. Any such belief would however be misleading. Indeed data indicate that the chief reason that patients are not cured of AML is that therapy (of any intensity) is ineffective rather than too toxic. This is true even in older patients. Thus, Appelbaum et al. reported that following administration of anthracycline + cytarabine (ara-C) in standard 3+7 fashion, resistance, defined as failure to enter CR despite not incurring TRM, was responsible for 71% of induction failures in patients age < 56, 61% in patients age 66–75, and 54% in patients age ≥ 75.^1^ Resistance also encompasses relapse from CR. Examining probabilities of relapse and death in remission in patients typically given high- or intermediate dose ara-C post remission Yanada et al. found ratios of relapse/death in CR of, as might be expected, 10–15/1 in patients age < 60 or 70 with performance status (PS) <2 at achievement of CR.^2^ However, even in patients aged ≥ 70 with PS 2–4 at this time, the ratio of relapse to death in CR was 3/1. AML not in remission has a very poor long-term prognosis. Hence, while development of less intense therapies is clearly important, emphasis should be placed on the effectiveness of a therapy rather than its “side effect profile” or “convenience”.

Furthermore, the term “intensive therapy”, while in common use, is inherently subjective. Here I will use it to describe therapies plausibly associated with an unacceptable probability of TRM or toxicity. Of course what constitutes “unacceptable” is also subject to debate and can only be discussed with reference to expected benefit from that treatment. For example a 10–15% risk of TRM from an intensive treatment such as high-dose cytarabine (ara-C) might be acceptable in a patient with “core-binding factor” (CBF) AML where the probability of long-term remission (CR) with high-dose ara-C (HiDAC) is > 50% and is very conceivably > 10–15% better than what be seen with a less intense therapy. In contrast the same 10–15% risk would be much less acceptable in a patient with a complex or monosomal karyotype where HiDAC is unlikely to produce a CR lasting more than 6–12 months.

These considerations aside, we do need a definition of intensive therapy. I think there would be little disagreement that doses of cytarabine (ara-C) ≥ 0.5 g/m2 fall into this category. Similarly there would be scant doubt that “low-dose ara-C”, or azacitidine and decitabine in usual doses and schedules are “non- intensive”. But how should we regard standard “3+7” or other similar treatments? For a healthy 40-year old 3+7, even with daunorubicin at 90mg/m2 daily X 3 days is probably non-intensive, but for an infirm 75 year old even a daunorubicin dose of 45mg/m2 might be intensive.

This emphasizes the need for consideration of which patients should be given less intense therapy. There appears to be a fair amount of disagreement surrounding this topic. For example, in their AML 16 trial in adults typically aged above 60, the MRC/NCRI group in the United Kingdom originally proposed a randomization between intensive and non-intensive therapy. However randomization occurred in < 5% of the patients who might have participated. Rather physicians decided that a given patient was better suited for intensive or non-intensive treatment. The principal determinant of assignment to intensive or non-intensive therapy was not age or performance (PS) but physician. Thus some physicians seemed more predisposed to offer intensive therapy and others more predisposed to non-intensive therapy.

As a consequence of this phenomenon the patients entered on a trial of one non-intensive therapy might not be entered on a trial of another non-intensive therapy. To me, the usefulness of any treatment can only be assessed in comparison to other treatments. Clearly the variability in patient selection criteria makes such comparison difficult absent randomization. I will return to this issue but for now suffice it to say that the great majority of phase 2 trials of non-intensive (or any) therapies are single-arm.

In any event, because standardization of selection criteria for assignment to non-intense therapy seems useful, this paper will begin with a discussion of this issue. We will next discuss results with azacitidine and decitabine, the two commonly employed non-intensive therapies. This will lead naturally to discussion of how these therapies might be improved and possible use of other non-intense therapies. We will conclude with discussion of trials involving such drugs might be conducted more efficiently.

## Selection of Patients for Less Intense Therapies

As noted above the decision to give a patient a less intense therapy depends on consideration of the ratio of benefit/risk with that therapy compared to a more intense therapy. Benefit relates to the probability that a given therapy would produce a long remission and would be assessed using covariates such as cytogenetics and molecular markers such as FLT3, NPM, CEBPA, DNMT3a etc.^3^

Risk relates most importantly to the probability of TRM. The distinction between TRM and resistance to therapy can be difficult. Because patients rarely obtain CR within 4 weeks of beginning therapy deaths occurring within that time might be said to constitute TRM. Estey et al.^4^ and subsequently Walter et al.^5^ approached this issue by looking at weekly death rates. They both found that once 4 weeks had elapsed from initiation of therapy, the probability of TRM declined sharply. This suggested that patients who died in these 4 weeks were qualitatively distinct and led to adoption of death within 28 days as a criterion for TRM.

It is intuitive to think of age as the primary determinant of TRM. Indeed eligibility for less intense treatment protocols typically requires that patients be older than 60–65. This practice implies that age is the principal determinant of TRM. One means to quantify the prognostic impact of a covariate uses areas under receiver operating characteristic curves (AUC).^5^ An AUC of 1.0 denotes that knowledge of a covariate leads to perfect prediction, for example as to whether a patient will incur 28-day TRM. In contrast, an AUC of 0.50 is the equivalent of a coin flip.

However examining data from 3,365 patients treated with 3+7 in Southwest Oncology Group (SWOG, 1127 patients median age 57) or with higher dose ara-C regimens at MD Anderson Cancer Center (MDA, 2238 patients median age 61) of whom 10% died by day 28, with similar proportions in SWOG and at MDA, Walter et al. found that the AUC for performance status (0.75) was higher than that for age (0.65).^5^ A model incorporating 8 covariates (performance status [PS], age, albumin, creatinine, WBC count, peripheral blood blast %, platelet count, and de novo vs. secondary AML) had an AUC of 0.82. Eliminating age from this model reduced the AUC for predicting 28-day TRM only slightly (0.82) (table 1). Thus prediction of 28-day TRM is enhanced by considering factors in addition to age, which is indeed a surrogate for many of these other factors. At Fred Hutchinson Cancer Center we employ a program, readily installed on an I-phone, to calculate a patient’s TRM score based on this 8-component model. The score is used to assign patients to treatment. For example patients with a score > 13.1, corresponding on average to a 31% risk of 28-day TRM, receive CPX 351, a liposomal combination of ara-C and daunorubicin at an “optimal molar ratio”, that is thought to be less toxic and perhaps more effective than standard 3+7.^6^,^7^

The analysis described in the preceding paragraph assumed that TRM rates are constant. However the past 10–15 years have seen the introduction of antifungal agents such as voriconazole, posaconazole, and itraconzaole as well other measures that might result in the ability to keep patients alive longer, thus affording more time for therapy to improve neutrophil counts and lessen the risk of infection. This prompted us to examine induction death rates as a function of time from 1991–2009.^8^ In particular, we analyzed these rates at 28 days (TRM) and at 60 days in 1409 SWOG patients (88% of whom received 3+7) and 1942 MDA patients, 92% of whom received ara-C at 1.5–2.0g/m2 daily X 3–5 days + other cytotoxic agents. TRM rates between 1991 and 2009 decreased from 18- 3% in SWOG and 16%- 4% at MDA. 60- day mortality rates decreased from 27% to 6% in SWOG and from 25% to 8% at MDA.^8^ Most of the decline in both TRM and 60-day mortality occurred from 2006–2009 (table 2). Multivariate analyses showed the decline was still present (p=0.001) after accounting for covariates associated with TRM, such as those noted in the preceding paragraph. Of course it is possible that the decline was merely a result of recent trends to give older patients less intense therapy. These patients were more likely “sicker” than the average patient. Hence the decline we observed might have resulted from selection bias. Arguing against this possibility however is that the decline in TRM rates occurred to approximately the same extent in younger and older patients and in patients with better and worse PS. It seems implausible that younger patients and/or those with better PS became more likely to receive less intense therapies from 2006–2009 than from 1991–2005. Hence it seems plausible that a real decline in TRM has occurred and should be taken into account when considering whether a patient should receive a more or a less intensive therapy.

## Azacitidine and Decitabine

In clinical practice in the United States azacitidine and decitabine are very probably the most commonly used low intensity therapies for patients with AML (> 20% blasts as specified by WHO)^9^ or higher-risk MDS, as defined for example by IPSS,^10^ IPSS-R,^11^ or WPSS.^12^ Because the natural history of higher risk MDS is closer to AML than to lower risk MDS and because groups such as MRC and HOVON and centers such as MDA and Fred Hutchinson allow patients with higher risk MDS as documented by > 10% blasts on AML trials, I will combine higher risk MDS and AML for this discussion. The focus will be on survival (OS).

Randomized trials have reported that either azacitidine or decitabine produce better OS than best supportive care (BSC) or low dose ara-C (LDAC) in patients with either higher risk MDS or AML or in patients with AML per se.^13^–^16^ In all these studies the drugs have reduced the risk of death to the same extent in higher risk MDS and in AML. However the azacitidine study included only AML patients with 21–30% blasts.^16^ An ongoing randomized trial (NCT01074047 at clinicaltrials.gov) is investigating the effects of azacitidine, BSC, and LDAC in patients with> 30% blasts.

Differences in OS reached statistical significance (p< 0.05) in the azacitidine trial^15^–^16^ but not in the decitabine trials.^13^–^14^ This reflected larger differences between azacitidine and BSC or LDAC than between decitabine and these treatments. Several factors must be kept in mind however before assuming azacitidine is the superior drug. First, the control group had better OS in the azacitidine study than in the decitabine studies. For example median OS with LDAC was 15–17 months in the former vs. 5 months in the latter. Indeed the difference between the LDAC groups in the azacitidine and the decitabine trials was greater than the difference between azacitidine and LDAC in the azacitidine study. This suggests that the control groups in azacitidine and decitabine trials differed considerably. The extent of selection bias is impossible to compare because while 0% of patients in the first^13^ and 23% of patients in the second azacitidine^14^ trials were considered for enrollment but not enrolled similar data were not provided for azacitidine. Second, while patients received a median of 4 cycles of decitabine they received a median of 8 cycles of azacitidine; of course this may have been a result rather than a cause of poor outcomes in some patients. Third, relatively few patients in the azacitidine studies and none in the decitabine studies received 3+7. However the hazard ratios favoring azacitidine have been less impressive when the drug is compared to 3+7 (0.76 and 0.97) rather than to BSC (0.58 and 0.48) or LDAC (0.36 and 0.37). MDA data indicate similar OS among 557 patients aged ≥ 65 treated with ara-C at doses of 1–2g/m2 +/− idarubicin or fludarabine +/− other drugs and 114 patients given decitabine (n=67) or azacitidine (n=47) despite higher CR rates with the more intense therapies, but the data are not derived from randomized trials.^17^

To me at least the fundamental question is not as much whether azacitidine or decitabine are better than BSC, LDAC, or 3+7 but rather whether the amount by which they are better justifies their frequent, at least in the U.S., designation as “standard of care”. US Social Security data indicate that a 65 -year old male will on average live another 18 years.^18^ The azacitidine data in indicate that a diagnosis of high risk MDS would result in a loss of 93% of this life expectancy with LDAC vs. 89% with azacitidine. Given these data I would expect some patients would prefer a clinical trial, possibly including azacitidine (or decitabine), to azacitidine alone. Hence while I agree that azacitidine and probably decitabine are standards to which newer therapies should be compared I believe that there is a clear need to investigate newer therapies (which might combine azacitidine or decitabine with other drugs) if significant medical, rather than merely statistical, progress is to be made.

It is generally accepted that allogeneic hematopoietic cell transplant (HCT) is currently needed to produce long-term remissions in AML or higher-risk MDS. A French study found no difference in post-HCT OS according to whether patients received azacitidine or intensive induction therapy pre-HCT.^19^ However it seems unlikely that azacitidine will be able to substitute for HCT if OS is the aim. Thus while recognizing the risks of selection bias and limiting their multivariate analysis to patients age 60–70 with high risk de novo MDS or secondary AML who either received HCT (n=105) or received azacitidine but not HCT(n=75) because of lack of a donor or institutional guidelines Platzbecker et al. found that after accounting for relevant prognostic variable HCT was associated with superior OS than azacitidine without HCT (2 year OS 39% vs. 23%).^20^

## Prediction of Response to Azacitidine or Decitabine

Although currently indispensable, randomized trials can be criticized for their focus on an average outcome. Thus while it seems likely that while the average patient will derive relatively little long-term improvement in OS from azacitidine or decitabine it is natural to ask whether we can identify patients who might benefit more than average.

Identification might involve clinical or biologic factors and these might be assessed pre- or post-treatment. Analyzing 282 consecutive patients with higher risk MDS or AML with 21–30% blasts given azacitidine and verifying their conclusions in an independent data set Itzykson et al. found that intermediate and poor risk cytogenetics, performance status ≥ 2, circulating blasts, and requirement for at least 4 units of RBCs / 8 weeks were independent predictors of shorter OS and could be used to define “low”, “intermediate”, and “high risk” groups with median OS of not reached, 15, and 6 months.^21^ However, only 10–15 % of patients were in the best group and the same factors might be expected to predict OS with therapies such as BSC, LDAC or 3+7. The same conclusion was reached by investigators who found that (a) chromosome 5 and/or 7 abnormalities and low hemoglobin and platelet levels were independently associated with shorter OS with decitabine,^22^ (b) the IPSS-R developed in patients who were untreated until they developed AML predicted outcome after azacitidine^23^ and (c) lack heterogeneity in benefit in different subgroups when comparing azacitidine to LDAC or BSC or azacitidine/decitabine to more “intensive” AML regimens.^15^,^16^

Examples of post treatment predictive clinical factors are number of courses administered and response to initial courses. It is often said that patients should receive at least 6 cycles of treatment before being declared resistant to azacitidine or decitabine. However, it has been reported that 82% of 50 patients who achieved CR, marrow CR (mCR) or “hematologic improvement” (HI) after receiving decitabine 20 mg/m2 daily X 5 had shown an initial response after 2 cycles and 90% after 3 cycles.^24^ In the oft-quoted azacitidine vs. BSC, LDAC, or 3+7 study^15^ median time to first response was 2 cycles with 80% of first responses seen by 4 cycles. About half of patients who achieve a response (CR, PR, HI) with azacitidine have been found have a better response after a median of another 3.5 cycles.^25^ However the relation between courses needed to achieve first or best response and subsequent OS or duration of response awaits further investigation.

Proper analysis of whether type of response (for example CR or < CR) affects OS requires a “landmark” analysis or one incorporating time dependent covariates to account for the time needed to achieve a given response. Such analyses have suggested that any response is associated with better OS than no response and that type of response (CR, CRp, PR, HI) is not so associated^26^ or that OS is similar as long as stable disease is obtained.^27^ Thus the need for CR to obtain longer OS, seen with traditional anti-AML therapy is unlikely to apply with azacitidine and decitabine; the same may apply with regard to survival beyond 2 years.^28^ Whether these relations would apply in patients who subsequently receive allogeneic hematopoietic cell transplant (HCT), which would often employ reduced intensity conditioning (RIC) remains to be seen; particularly given the general reluctance to perform RIC-HCT in patients with >5% marrow blasts.

## Azacitidine and Decitabine as “Hypomethylating Agents”

Although there is no doubt that these drugs affect hypomethylation due to their ability to inhibit DNA methyltransferase (DNMT) there appears to be more doubt regarding the importance of hypomethylation in clinical response. Certainly findings that the presence of DNMT mutations^29^ or higher levels of microRNA (miR)-29b which decrease levels of DNMT3 messenger RNA^30^ are associated with higher response rates with decitabine are consistent with a clinically relevant epigenetic mode of action for these drugs. Nonetheless it has proven difficult to consistently demonstrate correlations between response to azacitidine or decitabine and pre- or post-treatment DNA methylation status. The proclivity of different authors to analyze different genes complicates such attempts. Thus Shen et al. found no correlation between the pre-treatment methylation status of 10 genes and response to decitabine.^31^ However methylation averaged over multiple time points after therapy decreased more in patients given decitabine than BSC, and, in decitabine-treated patients, more with CR or PR than with HI and more with HI than with stable or progressive disease.^31^ Of course if, as suggested above, type of response does not affect OS the importance of post-treatment hypomethylation might become less. Ettou et al. found that low expression of FAS (a pro-apoptotic protein) at diagnosis, reflecting hypermethylation of the Fas gene promoter, correlated with response rate (CR+PR+HI) to azacitidine independently of age, IPSS, or prior therapy, although the derivation of the cutpoint used to define low Fas expression is not obvious and although while an increase in Fas expression after azacitidine correlated with response it did not correlate with OS.^32^ In patients given azacitidine + the histone deaetylase inhibitor etinostat and sequentially analyzing, during cycle 1, 4 presumed tumor suppressor genes, not including Fas but including 2 genes which were studied by Shen et al Fandy et al. could find no relation between clinical response, baseline methylation, or changes in methylation or gene expression in any of the 4 genes in either the bulk or the CD34 + population despite induction of histone acetylation.^33^ Klco et al. studied a vast number of genes 3 days after treatment with decitabine in clinically relevant concentrations and found only limited correlation between decitabine-induced changes in methylation and gene expression.^34^ Rather than affecting a specific group of genes, in particular those most commonly monitored after decitabine or azacitidine treatment, decitabine affected a range of genes, typically those most methylated pre treatment. The authors conclude: “the mechanism of action of decitabine is more complex than previously recognized”.^34^ The implications for rational development of epigenetic drugs are obvious.

## Newer Directions

- extended schedules of decitabine or azacitidine- although generally given at 20mg/m^2^ daily for 5 days, Blum et al. extended the duration to 10 days in 53 patents median age 74 with untreated AML. The CR rate was 47% and adding in responses < CR the response rates was 64%.^30^ Also using a 10-day schedule Ritchie et al. reported a CR rate of 40%.^35^ Although only about 10–12 months, median OS in these studies seems superior to that seen in the Kantarjian et al. randomized trial using 20mg/m^2^ daily X 5.^14^ All these patients are typically described as having “poor prognostic features”. Nonetheless it is not implausible that various biases might have been operating in each study, emphasizing the need for a randomized trial comparing the 5- and 10-day schedules. The same applies to reports that a 10- day schedule of azacitidine is superior to the conventional 7- day schedule.^36^
- azacitidine + lenalidomide – Sekeres et al. gave 36 patients, median age 68, with higher risk MDS azacitidine 75mg/m^2^ daily days 1–5 + lenalidomide 10 mg daily days 1–10 repeated every 28 days. While the CR rate was 44% vs. 17% in the Fenaux et al. study median OS was less than in the latter study^15^ (13 vs. 22 months), again pointing out the possible disconnect between CR and OS and stressing the need for the planned North American randomized trial comparing azcitidine +/− lenalidomide in MDS.
- newer less intense agents - the rapid entry into trials of many new drugs makes virtually any list of “new drugs in trial” incomplete and outdated. Perhaps the most prominent are inhibitors of various targets, often tyrosine kinases, thought to play a role in pathogenesis of AML. Examples include inhibitors of (a) FLT3 (such as quizartinib and crenolanib),(b) MDM2 thus restoring p53 activity (such as AMG (c) aminopeptidases (such as tosedostat) or (d) DNA repair (such as methoxyamine). The sheer number of new agents in trial suggests an uncertainty as to which is best. It is certainly plausible that because several aberrations exist in AML cells (or AML stem cells) agents focusing on one target will be insufficiently effective. However experience suggests that many agents that will ultimately not find a place in treatment will initially be called “promising” “encouraging” etc. Indeed several years ago we found that only 1 of 37 drugs thus considered after completion of early phase AML trials and reported as such at annual meetings of the American Society of Hematology (ASH) annual meetings had migrated into clinical practice despite a minimum follow-up of 5 years for each drug from date of presentation at ASH.^38^ This suggests a fundamental lesion in how we go about investigating new agents and we discuss this issue in the next section.
- issues with trials of new agents – these include small sample sizes and heterogeneity of patients included in trials. Endpoints are at times chosen without great regard for how they might affect OS, the outcome of most interest to patients; examples of such endpoints include responses such as hematologic improvement, CRi etc. Only rarely are we told the proportion of patients who although eligible for the trial were not treated on the trial. Given the well-known tendency of investigators to exclude patients who might do relatively poorly it would be appear that results of a trial that enrolled a higher proportion of eligible patients would more likely be reproducible. The unknown effect of such selection bias hampers the ability to compare one treatment with another. This is particularly problematic since often the most important question about a new agent is not what response rate or OS it produced but how these outcomes compare with those that might have been seen with other new agents or with standard therapy.

In the past comparison has been delayed until a phase 3 trial is organized and completed, often years after initial introduction of a new drug. This practice seems to be changing with the introduction of “selection” or “pick-a-winner” designs. These begin the process of comparison much earlier than previously and can involve either a new drug vs. a standard therapy (such as LDAC or in the future azacitidine or decitabine) or several new drugs vs. each other. These trials will obviously have considerable less power than the conventional phase 3 study. However the 80–90% power often specified in phase 3 trials is nominal because it ignores the process by which a new drug is chosen to compete against a standard. If we accept that, without comparative clinical data, it is very difficult to know which of many new therapies is best to compare to a standard and if there are, for example, 4 new agents that might be chosen as the investigational agent in a phase 3 trial, it follows that the chance of picking the best new agent is only 25%. Hence the true power of the phase 3 trial is 0.90 X 0.25, not 0.90. Selection designs indeed proceed under the assumption that the worse false negative results when a new drug is not investigated at all. Certainly selection/play-the-winner designs allow investigation, in a randomized setting, of more new drugs than possible using conventional methodology. Such designs are being increasingly frequently being used by European cooperative groups (e.g. MRC, HOVO, GIMEMA). They make it more difficult to identify a small subset that might benefit from a new drug. However if a response is seen in a given patient the trial could presumably close for all subsets except that represented by that patient.

## Conclusions

“Less intense” therapies are finding increasing application in AML. However it must be kept in mind that the principal problem in AML is not as much TRM (or toxicity) as it is lack of efficacy. Hence the principal goal of less intense therapies must be to improve efficacy not reduce toxicity. Assignment of patients to less intense therapies should be based on consideration of factors other than age, which is in any event not the most important predictor of TRM. Azacitidine and decitabine, the most commonly used less intense therapies in AML very probably produce better OS than BSC or LDAC. However improvement is relatively small when compared to expected life expectancy in the absence of disease. Accordingly, while azacitidine or decitabine should be considered the standards against which newer therapies are compared, continued investigation of potentially more effective therapies needs to continue. Better means for evaluating the large number of these therapies (and their combinations) are also needed.

## Figures and Tables

**Table 1:** Models predicting death within 28 days of start of induction therapy (TRM).

Model	Area under the curve (AUC)
Age alone	0.65
Performance status (PS) alone	0.75
Eight covariates including age and PS “maximal model”	0.83
Maximal model but without age	0.82

**Table 2:** Declining treatment-related mortality rates in SWOG and at MD Anderson

Cohort	Patients	1991–1995	1996–2000	2001–2005	2006–2009	P-value
SWOG	1409	18%	13%	12%	3%	<0.001
MDA	1942	16%	14%	9%	4%	<0.001

## References

[1] Appelbaum FR, Gundacker H, Head DR, Slovak ML, Willman CL, Godwin JE (2006). Age and acute myeloid leukemia. Blood.

[2] Yanada M G-MG, Borthakur G, Ravanadi F, Kantarjian H, Estey E (2008). Relapse and Death During First Remission in Acute Myeloid Leukemia. Haematologica.

[3] Dohner H, Estey EH, Amadori S, Appelbaum FR, Buchner T, Burnett AK Diagnosis and management of acute myeloid leukemia in adults: recommendations from an international expert panel, on behalf of the European LeukemiaNet. Blood.

[4] Estey E ST, Keating MJ, McCredie KB, Gehan EA, Freireich EJ (1989). Prediction of survival during induction therapy in patients with newly diagnosed acute myeloblastic leukemia. Leukemia.

[5] Walter RB OM, Borthakur G, Ravandi F, Cortes JE, Pierce SA, Appelbaum FR, Kantarjian HA, Estey EH (2011). Prediction of early death after induction therapy for newly diagnosed acute myeloid leukemia with pretreatment risk scores: a novel paradigm for treatment assignment. J Clin Oncol.

[6] Feldman EJ, Lancet JE, Kolitz JE, Ritchie EK, Roboz GJ, List AF First-in-man study of CPX-351: a liposomal carrier containing cytarabine and daunorubicin in a fixed 5:1 molar ratio for the treatment of relapsed and refractory acute myeloid leukemia. J Clin Oncol.

[7] Lancet JE, Cortes JE, Kovacsovics T, Hogge DE, Kolitz JE, Tallman MS (2012). CPX-351 Is Effective in Newly Diagnosed Older Patients with AML and with Multiple Risk Factors. ASH Annual Meeting Abstracts.

[8] Othus M, Kantarjian HM, Petersdorf S, Ravandi F, Cortes JE, Pierce SA (2012). Declining Rates of Treatment-Related Mortality in Patients with Newly Diagnosed Acute Myeloid Leukemia (AML) Given “Intensive” Induction Regimens: A Report From the Southwest Oncology Group (SWOG) and MD Anderson Cancer Center (MDA). ASH Annual Meeting Abstracts.

[9] Vardiman J, Thiele J, Arber D, Brunning R, Borowitz M, Porwit A (2009). The 2008 revision of the World Health Organization classification of myeloid neoplasms and acute leukemia:rationale and important changes. Blood.

[10] Greenberg P, Cox C, LeBeau MM, Fenaux P, Morel P, Sanz G (1997). International scoring system for evaluating prognosis in myelodysplastic syndromes. Blood.

[11] Greenberg PL, Tuechler H, Schanz J, Sanz G, Garcia-Manero G, Sole F (2012). Revised international prognostic scoring system for myelodysplastic syndromes. Blood.

[12] Malcovati L, Germing U, Kuendgen A, Della Porta MG, Pascutto C, Invernizzi R (2007). Time-dependent prognostic scoring system for predicting survival and leukemic evolution in myelodysplastic syndromes. J Clin Oncol.

[13] Lubbert M, Suciu S, Baila L, Ruter BH, Platzbecker U, Giagounidis A (2011). Low-dose decitabine versus best supportive care in elderly patients with intermediate- or high-risk myelodysplastic syndrome (MDS) ineligible for intensive chemotherapy: final results of the randomized phase III study of the European Organisation for Research and Treatment of Cancer Leukemia Group and the German MDS Study Group. J Clin Oncol.

[14] Kantarjian HM, Thomas XG, Dmoszynska A, Wierzbowska A, Mazur G, Mayer J (2012). Multicenter, Randomized, Open-Label, Phase III Trial of Decitabine Versus Patient Choice, With Physician Advice, of Either Supportive Care or Low-Dose Cytarabine for the Treatment of Older Patients With Newly Diagnosed Acute Myeloid Leukemia. Journal of Clinical Oncology.

[15] Fenaux P, Mufti GJ, Hellstrom-Lindberg E, Santini V, Finelli C, Giagounidis A (2009). Efficacy of azacitidine compared with that of conventional care regimens in the treatment of higher-risk myelodysplastic syndromes: a randomised, open-label, phase III study. Lancet Oncol.

[16] Fenaux P, Mufti GJ, Hellstrom-Lindberg E, Santini V, Gattermann N, Germing U (2010). Azacitidine Prolongs Overall Survival Compared With Conventional Care Regimens in Elderly Patients With Low Bone Marrow Blast Count Acute Myeloid Leukemia. Journal of Clinical Oncology.

[17] Quintas-Cardama A, Ravandi F, Liu-Dumlao T, Brandt M, Faderl S, Pierce S (2012). Epigenetic therapy is associated with similar survival compared with intensive chemotherapy in older patients with newly diagnosed acute myeloid leukemia. Blood.

[b18-mjhid-5-1-e2013050] 18Accessed at http://www.socialsecurity.gov/OACT/population/longevity.html

[19] Damaj G, Duhamel A, Robin M, Beguin Y, Michallet M, Mohty M (2012). Impact of azacitidine before allogeneic stem-cell transplantation for myelodysplastic syndromes: a study by the Societe Francaise de Greffe de Moelle et de Therapie-Cellulaire and the Groupe-Francophone des Myelodysplasies. J Clin Oncol.

[20] Platzbecker U, Schetelig J, Finke J, Trenschel R, Scott BL, Kobbe G (2012). Allogeneic hematopoietic cell transplantation in patients age 60–70 years with de novo high-risk myelodysplastic syndrome or secondary acute myelogenous leukemia: comparison with patients lacking donors who received azacitidine. Biol Blood Marrow Transplant.

[21] Itzykson R, Thepot S, Quesnel B, Dreyfus F, Beyne-Rauzy O, Turlure P (2011). Prognostic factors for response and overall survival in 282 patients with higher-risk myelodysplastic syndromes treated with azacitidine. Blood.

[22] Jabbour E, Garcia-Manero G, Ravandi F, Faderl S, O’Brien S, Fullmer A (2013). Prognostic factors associated with disease progression and overall survival in patients with myelodysplastic syndromes treated with decitabine. Clin Lymphoma Myeloma Leuk.

[23] Lamarque M, Raynaud S, Itzykson R, Thepot S, Quesnel B, Dreyfus F (2012). The revised IPSS is a powerful tool to evaluate the outcome of MDS patients treated with azacitidine: the GFM experience. Blood.

[24] Steensma DP, Baer MR, Slack JL, Buckstein R, Godley LA, Garcia-Manero G (2009). Multicenter study of decitabine administered daily for 5 days every 4 weeks to adults with myelodysplastic syndromes: the alternative dosing for outpatient treatment (ADOPT) trial. J Clin Oncol.

[25] Silverman LR, Fenaux P, Mufti GJ, Santini V, Hellstrom-Lindberg E, Gattermann N (2011). Continued azacitidine therapy beyond time of first response improves quality of response in patients with higher-risk myelodysplastic syndromes. Cancer.

[26] Thepot S, Itzykson R, Seegers V, Recher C, Quesnel B, Delaunay J (2009). Azacytidine (AZA) as First Line Therapy in AML: Results of the French ATU Program. ASH Annual Meeting Abstracts.

[27] Gore S, Fenaux P, Santini V, Bennett JM, Silverman LR, Seymour JF (2010). Time-dependent decision analysis: Stable disease in azacitidine (AZA)-treated patients (pts) with higher-risk MDS. J Clin Oncol.

[28] Itzykson R, Thepot S, Beyne-Rauzy O, Quesnel B, Ame S, Turlure P (2010). Prolonged Survival without Complete Remission (CR) In AML Patients (Pts) Treated with Azacitidine (AZA). ASH Annual Meeting Abstracts.

[29] Metzeler KH, Walker A, Geyer S, Garzon R, Klisovic RB, Bloomfield CD (2012). DNMT3A mutations and response to the hypomethylating agent decitabine in acute myeloid leukemia. Leukemia.

[30] Blum W, Garzon R, Klisovic RB, Schwind S, Walker A, Geyer S (2010). Clinical response and miR-29b predictive significance in older AML patients treated with a 10-day schedule of decitabine. Proceedings of the National Academy of Sciences.

[31] Shen L, Kantarjian H, Guo Y, Lin E, Shan J, Huang X (2009). DNA methylation predicts survival and response to therapy in patients with myelodysplastic syndromes. J Clin Oncol.

[32] Ettou S, Audureau E, Humbrecht C, Benet B, Jammes H (2012). Fas expression at diagnosis as a biomarker of azacitidine activity in high-risk MDS and secondary AML. Leukemia.

[33] Fandy TE, Herman JG, Kerns P, Jiemjit A, Sugar EA, Choi S-H (2009). Early epigenetic changes and DNA damage do not predict clinical response in an overlapping schedule of 5-azacytidine and entinostat in patients with myeloid malignancies. Blood.

[34] Klco JM, Spencer DH, Lamprecht TL, Sarkaria SM, Wylie T, Magrini V (2013). Genomic impact of transient low-dose decitabine treatment on primary AML cells. Blood.

[35] Ritchie EK, Feldman EJ, Christos PJ, Rohan SD, Lagassa CB, Ippoliti C Decitabine in patients with newly diagnosed and relapsed acute myeloid leukemia. Leuk Lymphoma.

[36] Prebet T, Sun Z, Ketterling R, Hicks G, Beach CL, Greenberg PL (2010). A 10 Day Schedule of Azacitidine Induces More Complete Cytogenetic Remissions Than the Standard Schedule In Myelodysplasia and Acute Myeloid Leukemia with Myelodysplasia-Related Changes: Results of the E1905 US Leukemia Intergroup Study. ASH Annual Meeting Abstracts.

